# The function of cux1 in oxidative dna damage repair is needed to prevent premature senescence of mouse embryo fibroblasts

**DOI:** 10.18632/oncotarget.2919

**Published:** 2015-02-17

**Authors:** Zubaidah M. Ramdzan, Ranjana Pal, Simran Kaur, Lam Leduy, Ginette Bérubé, Sayeh Davoudi, Charles Vadnais, Alain Nepveu

**Affiliations:** ^1^ Goodman Cancer Centre, McGill University, Montreal, Quebec H3A 1A3, Canada; ^2^ Department of Biochemistry, McGill University, Montreal, Quebec H3A 1A3, Canada; ^3^ Department of Medicine, McGill University, Montreal, Quebec H3A 1A3, Canada; ^4^ Department of Oncology, McGill University, Montreal, Quebec H3A 1A3, Canada; ^5^ Department of Biological Sciences, Presidency University, Kolkata 700073, India

**Keywords:** cellular senescence, mouse embryo fibroblasts, oxidative DNA damage, 8-oxoguanine DNA glycosylase 1 (OGG1), CUX1

## Abstract

Despite having long telomeres, mouse embryo fibroblasts (MEFs) senesce more rapidly than human diploid fibroblasts because of the accumulation of oxidative DNA damage. The CUX1 homeodomain protein was recently found to prevent senescence in RAS-driven cancer cells that produce elevated levels of reactive-oxygen species. Here we show that Cux1^−/−^ MEFs are unable to proliferate in atmospheric (20%) oxygen although they can proliferate normally in physiological (3%) oxygen levels. CUX1 contains three domains called Cut repeats. Structure/function analysis established that a single Cut repeat domain can stimulate the DNA binding, Schiff-base formation, glycosylase and AP-lyase activities of 8-oxoguanine DNA glycosylase 1, OGG1. Strikingly and in contrast to previous reports, OGG1 exhibits efficient AP-lyase activity in the presence of a Cut repeat. Repair of oxidative DNA damage and proliferation in 20% oxygen were both rescued in Cux1^−/−^ MEFs by ectopic expression of CUX1 or of a recombinant Cut repeat protein that stimulates OGG1 but is devoid of transcription activation potential. These findings reinforce the causal link between oxidative DNA damage and cellular senescence and suggest that the role of CUX1 as an accessory factor in DNA repair will be critical in physiological situations that generate higher levels of reactive oxygen species.

## INTRODUCTION

The presence of senescent cells is associated with, and is believed to contribute to, many age-related pathologies [1, 2]. Cellular senescence is triggered by many types of genomic damage, including telomere shortening and oxidative DNA damage [3]. For example, MEFs have long telomeres and express telomerase but senesce more rapidly than human diploid fibroblasts because of the accumulation of oxidative DNA damage [4]. MEFs become senescent after 4 to 5 weeks when cultured in 20% oxygen, but proliferate indefinitely when maintained in 3% oxygen [4]. Moreover, MEFs cultured in 3% oxygen for 10–15 population doublings eventually undergo a mutagenic or adaptive event that allows them to proliferate in 20% oxygen [4].

Reactive oxygen species (ROS) produced by external sources or generated through cellular metabolism represent a major threat to the integrity of DNA. Among the most deleterious of ROS-induced adducts in DNA is 7,8-dihydro-8-oxoguanine (8-oxoG), which can mispair with adenine and cause G-C to T-A transversion mutations [5]. 8-oxoG lesions are removed by base excision repair, a process that is initiated by specific DNA glycosylases. In human cells, oxidative purine lesions are removed by 8-oxoguanine DNA glycosylase 1, OGG1, whereas oxidative pyrimidine lesions are removed by NTH1, NEIL1, or NEIL2 (reviewed in [6]). DNA glycosylases for oxidized bases are bifunctional. These enzymes hydrolyze the N-glycosidic bond to generate an apurinic/apyridinic (AP) site and then generate a single-strand nick 3′ to the AP site via beta (OGG1, NTH1) or beta-delta (NEIL1, NEIL2) elimination. End-processing of the resulting single-strand break is then performed by APE1 or PNKP, and repair synthesis and ligation are accomplished by the short-patch or long-patch pathways [7, 8].

Deficient repair of oxidative DNA damage has been implicated in both cancer development and neurodegenerative disorders [9]. Functional analysis of DNA repair enzymatic activity showed that reduced activity of OGG1 is a risk factor in lung and head and neck cancer [10, 11]. The regulation of OGG1 activity may occur through a variety of mechanisms including transcriptional control, protein-protein interactions, posttranslational modifications and dynamic localization, however, none of these mechanisms can explain the observed differences among individuals [12]. The existence of single nucleotide polymorphisms in the human OGG1 gene has triggered a number of epidemiological studies to evaluate the association with sporadic cancers. Overall, these studies led to conflicting results and suggest a minor effect at best [13].

The Cut homeobox 1 gene, *CUX1*, has been implicated in both tumor suppression and tumor progression. While CUX1 is the target of monoallelic deletion or inactivating somatic mutations in many cancers, increased CUX1 expression is associated with shorter disease-free survival (reviewed in [14]). Indeed, transgenic mice expressing specific CUX1 isoforms develop tumors or cancer-associated disorders in various tissues [15–21]. The biochemical and cellular functions of CUX1 involved in tumor suppression and/or progression remain to be fully characterized. CUX1 encodes two main isoforms with strikingly different properties (reviewed in [22, 23]). The proteolytically processed p110 CUX1 isoform makes a stable interaction with DNA and functions as a transcriptional repressor or activator depending on the promoter context [24–26]. Much less is known about the biochemical and cellular functions of the full-length protein, p200 CUX1. This isoform is very abundant and binds DNA with extremely fast kinetics (rapid “on” and “off” rates), using four evolutionarily conserved DNA binding domains: three Cut repeats (CR1, CR2 and CR3) and a Cut homeodomain (HD) [27]. These properties are not consistent with a role as a classical transcription factor that binds stably to a limited number of genomic sites where it recruits a co-activator or a co-repressor. Indeed, we have recently uncovered a direct role of p200 CUX1 in DNA repair [16]. Knockdown or genetic inactivation of CUX1 delays, whereas ectopic p200 CUX1 expression, accelerates repair of DNA damage. *In vitro*, recombinant CUX1 proteins stimulate the glycosylase activity of OGG1 on oligonucleotides containing an 8-oxoguanine. Elevated CUX1 expression prevents senescence caused by a RAS oncogene in human primary cells and cooperates with RAS in promoting tumorigenicity in the mouse [16]. As CUX1 knockdown is synthetic lethal for cells that harbor a RAS oncogene [16, 28], these findings established a case of non-oncogene addiction whereby RAS-driven cancer cells have become acutely dependent on the heightened expression and activity of a normal protein, p200 CUX1, that is not itself oncogenic (reviewed in [29, 30]).

In the present study, we used mouse embryo fibroblasts derived from a Cux1^−/−^ knockout mouse to investigate the physiological role of CUX1 in oxidative DNA damage repair. Proliferation assays indicate that CUX1 is required for cells to avoid senescence and continue to proliferate in the presence of oxidative stress. Structure-function analysis and *in vitro* assays with purified components established that a single Cut repeat domain is sufficient to stimulate many biochemical activities of OGG1 including DNA binding, Schiff-base formation, glycosylase and AP-lyase reactions.

## RESULTS

### Genetic inactivation of Cux1 causes a proliferation block in atmospheric (20%) oxygen

**Figure 1:**
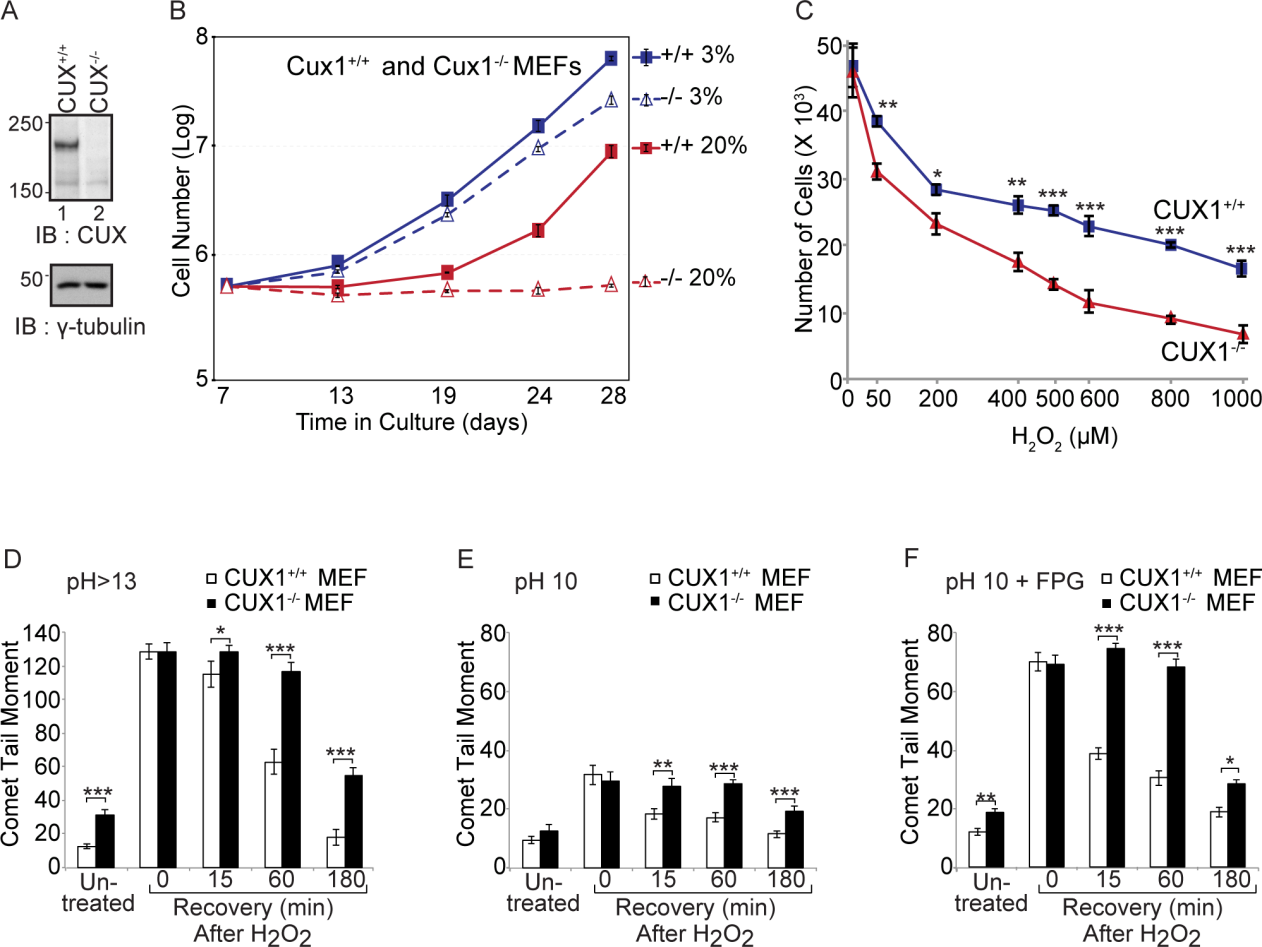
Genetic inactivation of Cux1 causes a proliferation block in atmospheric (20%) oxygen **(A)** Immunoblotting analysis using CUX1–1300 antibody. **(B)** Cux1^+/+^ and Cux1^−/−^ MEFs were cultured in 3% or 20% oxygen and counted over a period of 21 days. **(C)** Cux1^+/+^ and Cux1^−/−^ MEFs were maintained in 3% oxygen for 7 days and exposed to various concentration of H_2_O_2_ for 60 min and then cultured in fresh medium for 24 h in 3% oxygen before counting the cells. Error bars represent standard error. *p < 0.05, **p < 0.01, ***p < 0.001; Student's *t*-test. **(D, E and F)** Cux1^+/+^ and Cux1^−/−^ MEFs were maintained in 3% oxygen for 7 days, exposed to 50 μM H_2_O_2_ for 20 minutes and allowed to recover for the indicated time. Cells were submitted to single cell gel electrophoresis at pH > 13 **(D)**, pH 10 **(E)**, and pH 10 in the presence of FPG **(F)** Comet tail moments were scored for at least 50 cells per condition. Error bars represent standard error. *p < 0.05, **p < 0.01, ***p < 0.001; Student's *t*-test.

Since the perinatal lethality of Cux1^−/−^ knockout mice precludes further phenotypic analysis, we employed mouse embryo fibroblasts (MEFs) to investigate the consequences of CUX1 inactivation (Figure 1A). We compared the proliferative capacity of Cux1^+/+^ and Cux1^−/−^ MEFs in 3% and 20% oxygen. While Cux1^−/−^ MEFs proliferated slightly more slowly than Cux1^+/+^ MEFs in 3% oxygen, they exhibited a drastic proliferation defect in 20% oxygen (Figure 1B). The striking proliferation block in 20% oxygen suggested that Cux1^−/−^ MEFs were sensitive to oxidative stress. Indeed, Cux1^−/−^ MEFs exhibited hypersensitivity to treatment with increasing concentrations of H_2_O_2_ (Figure 1C). We therefore compared the capacity of these cells to repair oxidative DNA damage. Cux1^+/+^ and Cux1^−/−^ MEFs, maintained in 3% oxygen for 7 days, were treated with H_2_O_2_ and submitted to single cell gel electrophoresis (comet assay) after variable recovery periods. Comet assays performed at pH > 13 showed that the repair of oxidative DNA damage is delayed in Cux1^−/−^ MEFs (Figure 1D). Comet assays in these alkaline conditions (pH > 13) detect double-strand and single-strand breaks as well as abasic sites and several types of altered bases that are intrinsically labile at high pH. In contrast, comet assays performed at pH 10 only detects double-strand breaks and single-strand breaks (Figure 1E). Addition of the formamidopyrimidine DNA glycosylase (FPG) allows the detection of most types of oxidized bases, including 8-oxoG and formamidopyrimidines. Treatment with FPG indicated that the repair of oxidized bases is delayed in Cux1^−/−^ MEFs, pointing to a specific defect in base excision repair, particularly in the repair of oxidized bases (Figure 1F).

### A recombinant CUX1 protein that is devoid of transcriptional activity can prevent the accumulation of oxidative DNA damage

**Figure 2:**
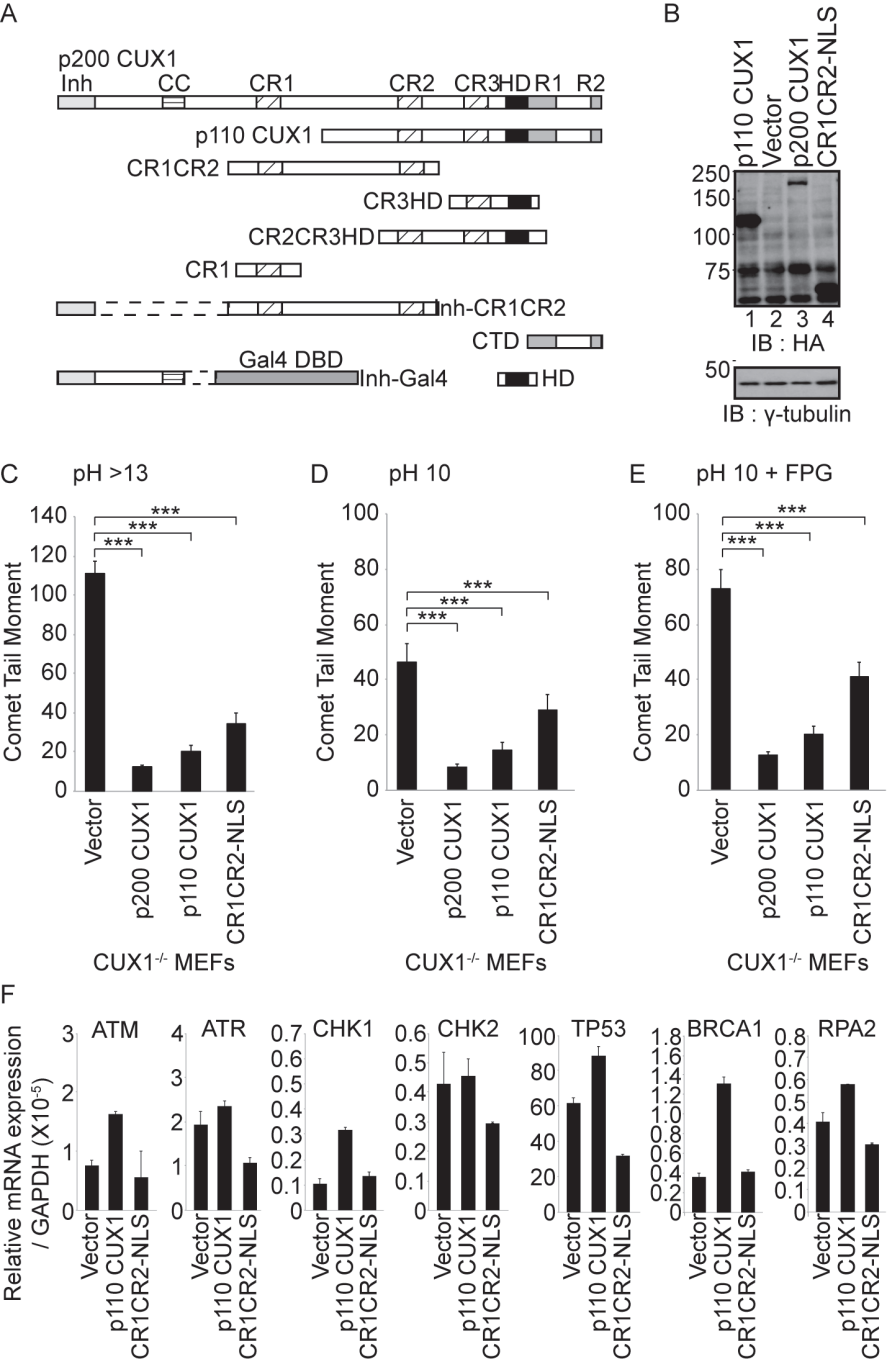
Rescue of DNA repair defect by recombinant CUX1 proteins **(A)** Schematic representation of CUX1 proteins used in this study. Note that the proteins expressed in bacteria contain a histidine tag at their N-terminus. The CR1CR2 expressed in mammalian cells contains in addition a nuclear localization signal at its C-terminus. Shown at the top are the functional domains: Inh, auto-inhibitory domain; CC, coiled-coil; CR1, CR2 and CR3, Cut repeat 1, 2 and 3; HD, Cut homeodomain; R1 and R2, repression domains 1 and 2. Note that the auto-inhibitory domain prevents binding by covalently linked DNA binding domains [35]. **(B)** Cux1^−/−^ MEFs were cultured in 3% oxygen and stably infected with retroviruses expressing p200 CUX1-HA, p110 CUX1-HA, CR1CR2-NLS-HA or nothing (vector). Expression of recombinant CUX1 protein expression was analyzed by immunoblotting using HA antibodies. **(C, D and E)** Comet assays were performed after 32 days of culture in three conditions as described in Figure 1. Comet tail moments were scored for at least 50 cells per conditions. Error bars represent standard error. ***p < 0.001; Student's *t*-test. **(F)** mRNA levels of transcriptional targets of CUX1 involved in DNA damage responses. All mRNA levels were normalized to glyceraldehyde 3-phosphate dehydrogenase (GAPDH). The values are the mean of three measurements and error bars represent standard deviation.

Cux1^−/−^ MEFs carrying an empty vector and maintained in 3% oxygen can proliferate but gradually accumulate oxidative DNA damage, as revealed by comet assays performed on day 32 (Figure 2B, and 2E; compare with comet assays of untreated cells in Figure 1D, and 1F). DNA damage, however, was greatly reduced by ectopic expression of p200 or p110 CUX1, the main two isoforms of CUX1 (Figure 2A and 2E). The increase in DNA repair capacity conferred by CUX1 expression could involve a transcriptional or a non-transcriptional role of CUX1 in DNA repair, since the p110 CUX1 isoform has previously been shown to activate the expression of many genes involved in DNA damage responses [31]. To examine the possibility of a non-transcriptional role of CUX1 in DNA repair, we engineered a retroviral vector to express a recombinant protein encompassing the Cut repeats 1 and 2 fused to a nuclear localization signal, CR1CR2-NLS (see map in Figure 2A). This protein exhibits very fast DNA binding kinetics and lacks the amino acids required for transcriptional activation [27, 32]. Indeed, gene expression analysis confirmed that transcriptional targets of p110 CUX1 that are involved in DNA damage responses were not upregulated in Cux1^−/−^ MEFs stably expressing CR1CR2-NLS (Figure 2F). Yet, CR1CR2-NLS reduced DNA damage in MEFs despite its inability to activate transcription (Figure 2C, and 2E). These findings suggest that CUX1 may play a direct role in the repair of oxidized bases in addition to its role as a transcriptional activator of DNA damage response genes [31].

### CUX1 knockdown reduces the 8-oxoG cleavage activity of cell extract

**Figure 3:**
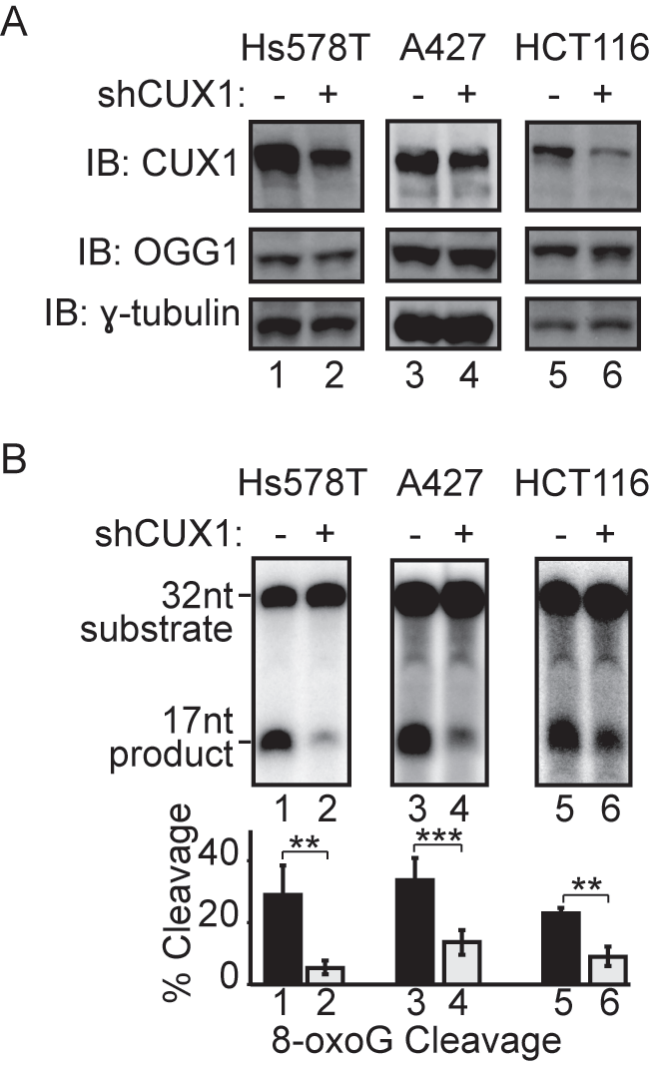
CUX1 knockdown reduces the 8-oxoG cleavage activity of human cell lines Lentivirus expressing a doxycycline inducible shRNA against CUX1 was introduced in Hs578T, A427 and HCT116 cell lines. Doxycycline was added to the medium, total protein extracts were prepared after 4 days and used in immunoblotting analysis using the indicated antibodies **(A)** and 8-oxoG cleavage assays **(B)**. The assays were conducted using 20 μg of total proteins from each cell line and radiolabeled double-stranded oligonucleotides containing an 8-oxoG or a normal guanine base (see probe A in Figure S1A). Percentages of cleaved product from 3 independent experiments are shown below. Error bars represent standard error using Student's t-test, ***p < 0.001; **p < 0.01. From image scanning analysis, the extent of knockdown and cleavage inhibition were 88% and 81% in Hs578T, 35% and 58% in A427, and 68% and 60% in HCT116 cells.

Since 8-oxoG is the most abundant DNA adduct following treatment with an oxidizing agent, we investigated the effect of CUX1 knockdown on the efficiency of cell extracts to process oligonucleotides containing an 8-oxoG base (see probe A in Figure S3A). Three independent cell lines were infected with a lentivirus expressing a doxycycline-inducible shRNA against CUX1. Upon treatment with doxycycline, CUX1 protein expression was substantially reduced in all 3 cell lines, but OGG1 levels remained unchanged (Figure 3A). In each of the three cell lines, CUX1 knockdown significantly reduced the efficiency of 8-oxoG removal *in vitro* (Figure 3B, lanes 2, 4 and 6).

### Interaction between CUX1 and OGG1

**Figure 4:**
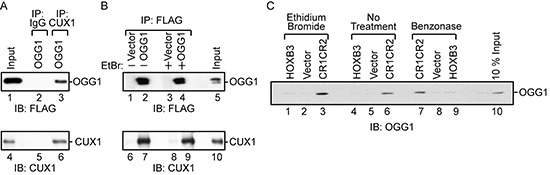
Interaction between OGG1 and CUX1 **(A and B)** 293T cells were transfected with CUX1 (CR2CR3HD; map in Figure 2A), FLAG-OGG1 or the empty vector, as indicated. **(A)** Total protein extracts were loaded on gel (input) or were submitted to immunoprecipitation with the indicated antibodies (Preimmune; IgG or CUX1) and then immunoblotted with FLAG and CUX1 antibodies. **(B)** Total protein extracts were submitted to immunoprecipitation with Flag antibodies in the absence or presence of ethidium bromide. **(C)** A pull-down assay was performed using purified GST-OGG1 and either beads bound to his-tagged CUX1-CR1CR2, HOXB3 or vector alone, followed by immunoblotting with anti-OGG1 in the presence and absence of ethidium bromide or after treatment of protein samples with benzonase.

Co-immunoprecipitation experiments demonstrated that CUX1 is able to form a complex with FLAG-tagged OGG1 in cells. The FLAG-OGG1 protein was detected by immunoblotting following immunoprecipitation with CUX1 antibodies (Figure 4A, lane 3). Reciprocally, the CUX1 protein was detected by immunoblotting following immunoprecipitation with FLAG antibodies in the presence and absence of ethidium bromide (Figure 4B, lane 7 and lane 9). Pull-down assays using purified GST-tagged OGG1 and a his-tagged CUX1 protein containing the CR1 and CR2 domains established that the two proteins were able to engage in a direct interaction (Figure 4C, lane 6). Importantly, the interaction was maintained when the assay was performed in the presence of ethidium bromide or benzonase (Figure 4C, lanes 3 and 7).

### Cut repeats stimulate the glycosylase and AP-lyase activities of OGG1 *in vitro*

**Figure 5:**
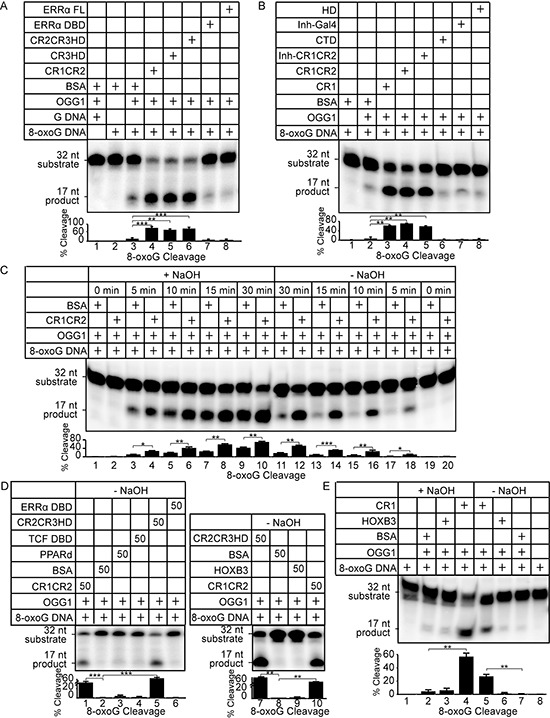
Cut repeats stimulate the glycosylase and AP-lyase activities of OGG1 Double-stranded oligonucleotides containing an 8-oxoG or an unmodified G were radioactively end-labeled and used in cleavage and DNA binding assays. Sequences of probe A substrate and product are shown in Figure S1A. Percentages of cleaved product from 3 independent experiments are shown below each lane. Error bars represent standard error using Student's t-test, ***p < 0.001; **p < 0.01; *p < 0.05. **(A and B)** The 8-oxoG cleavage assay was performed for 30 min. using OGG1 (New England Biolabs, Ipswich, MA) and bacterially purified proteins as indicated. A diagrammatic representation of CUX1 proteins is shown in Figure 2A: CR1, CR1CR2, Inh-CR1CR2, CR2CR3HD, CR3HD, HD, CTD. Coomassie stains are presented in Figure S1B. ERRα FL is the full length estrogen-related receptor protein; ERRαDBD, the ERRα DNA binding domain; Inh-Gal4, the yeast Gal4 transcription factor fused to the CUX1 auto-inhibitory domain. **(C)** The 8-oxoG cleavage assay was performed using 50 nM OGG1 and bacterially purified CUX1 CR1CR2. After 0, 5, 10, 15, 30 min incubation at 37°C, reactions were stopped, and DNA was submitted to treatment with NaOH (+NaOH) or not (−NaOH). Reactions in the presence of NaOH monitor OGG1 glycosylase activity only, whereas reactions in the absence of NaOH reveal OGG1 glycosylase and AP-lyase activities. **(D)** The 8-oxoG cleavage assay was performed for 30 min. with OGG1 and the indicated proteins and was stopped without treatment with NaOH. **(E)** The 8-oxoG cleavage assay was performed using 50 nM OGG1 and bacterially purified CUX1 CR1. The reaction was incubated for 30 min at 37°C and the DNA was treated either NaOH (+NaOH) or not (−NaOH) prior to migration on the gel.

Using 8-oxoG cleavage assays with purified proteins, we previously showed that various combinations of Cut repeats and the Cut homeodomain (CR1CR2, CR3HD and CR2CR3HD) are able to stimulate the glycosylase activity of OGG1 [16]. Since each of these recombinant proteins is able to bind to DNA with high affinity, a logical conclusion from these experiments is that high affinity DNA binding contributes to the stimulation of OGG1. Here, we performed structure/function analysis to test this notion and we further characterized the effect of CUX1 on distinct biochemical activities of OGG1. The glycosylase activity of OGG1 was stimulated in the presence of any recombinant CUX1 protein that contains one or more Cut repeats: CR1CR2, CR3HD, CR2CR3HD and CR1 (Figure 5A, compare lane 3 with 4, 5 and 6; Figure 5B, compare lane 2 with 3, 4 and 5). The stimulation of OGG1 appears to be specific to the Cut repeat domain since there was no effect of other regions of the CUX1 protein, including the carboxy-terminal domain (CTD) and the Cut homeodomain (HD) (Figure 5B, lanes 6 and 8). Also, we observed no stimulation by other DNA binding proteins, including the full-length estrogen-related receptor alpha (ERR-FL), the ERR DNA binding domain (ERR-DBD) or the Gal4 DNA binding domain (Figure 5A, lanes 7–8; 5B, lane 7). Electrophoretic mobility shift assays (EMSAs) confirmed that the ERR-FL and ERR-DBD proteins were folded properly since they were able to bind to their cognate DNA binding site (Figure S2). Increasing the concentration of Cut repeat proteins led to increased OGG1 glycosylase activity (Figure 3SA, probe A; Figure S3B, cleavage assay).

The glycosylase and AP-lyase activities of OGG1 can be distinguished by incubating the oligonucleotides with or without NaOH at the end of the reaction. A time-course comparison of the products generated in the presence of NaOH shows that OGG1 alone generates an increasing amount of abasic sites that are cleaved by alkali treatment (Figure 5C, lanes 3, 5, 7, 9). In the absence of NaOH, only a small fraction of these abasic sites are converted to a single-strand break by the AP-lyase activity of OGG1 (Figure 5C, lanes 17, 15, 13, 11). These results are in agreement with previous studies reporting a weak AP-lyase activity of OGG1 on its own [33]. When reactions were carried out with CR1CR2 and OGG1, however, the amount of cleaved products in the presence or absence of NaOH were comparable indicating that the AP-lyase activity of OGG1 is stimulated by CR1CR2 (Figure 5C, compare lanes 4 and 18, 6 and 16, 8 and 14, 10 and 12). Increasing the concentration of CR1CR2 led to higher OGG1 AP-lyase activity (Figure S3C, right panel). The ability of CR1CR2 to stimulate the glycosylase and AP-lyase activities of OGG1 was confirmed using oligonucleotides with a different DNA sequence (Figure S4). CR2CR3HD was also able to stimulate the AP-lyase activity of OGG1 (Figure 5D, compare lanes 5 and 2, 7 and 8). In contrast to Cut repeats, HOXB3, TCF-DBD, PPARδ and the DNA binding domain of ERRα had very weak or no effect on the AP-lyase activity of OGG1 (Figure 5D, compare lane 2 with 3, 4, and 6; and 8 with 9). Importantly, CR2CR3HD and CR1CR2 were unable alone to cleave a probe containing an 8-oxoG residue or an abasic site (Figure S5A and S5B).

### Cut repeats stimulate the formation of a Schiff-base intermediate and the binding of OGG1 to 8-oxoG DNA

**Figure 6:**
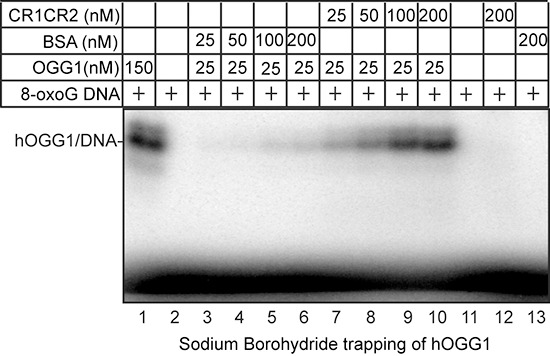
Sodium borohydride trapping of OGG1 enzyme in the presence of CR1CR2 5′-end-labeled 32-mer duplex containing an 8-oxoG was incubated with hOGG1 and CR1CR2 or BSA at the indicated concentrations. After incubation at 37°C, 50 mM sodium borohydride was added. The reactions were pursued for another 5 min at 37°C. After termination of the reaction, the trapped complexes were separated from free substrate by 10% SDS-PAGE gel.

**Figure 7:**
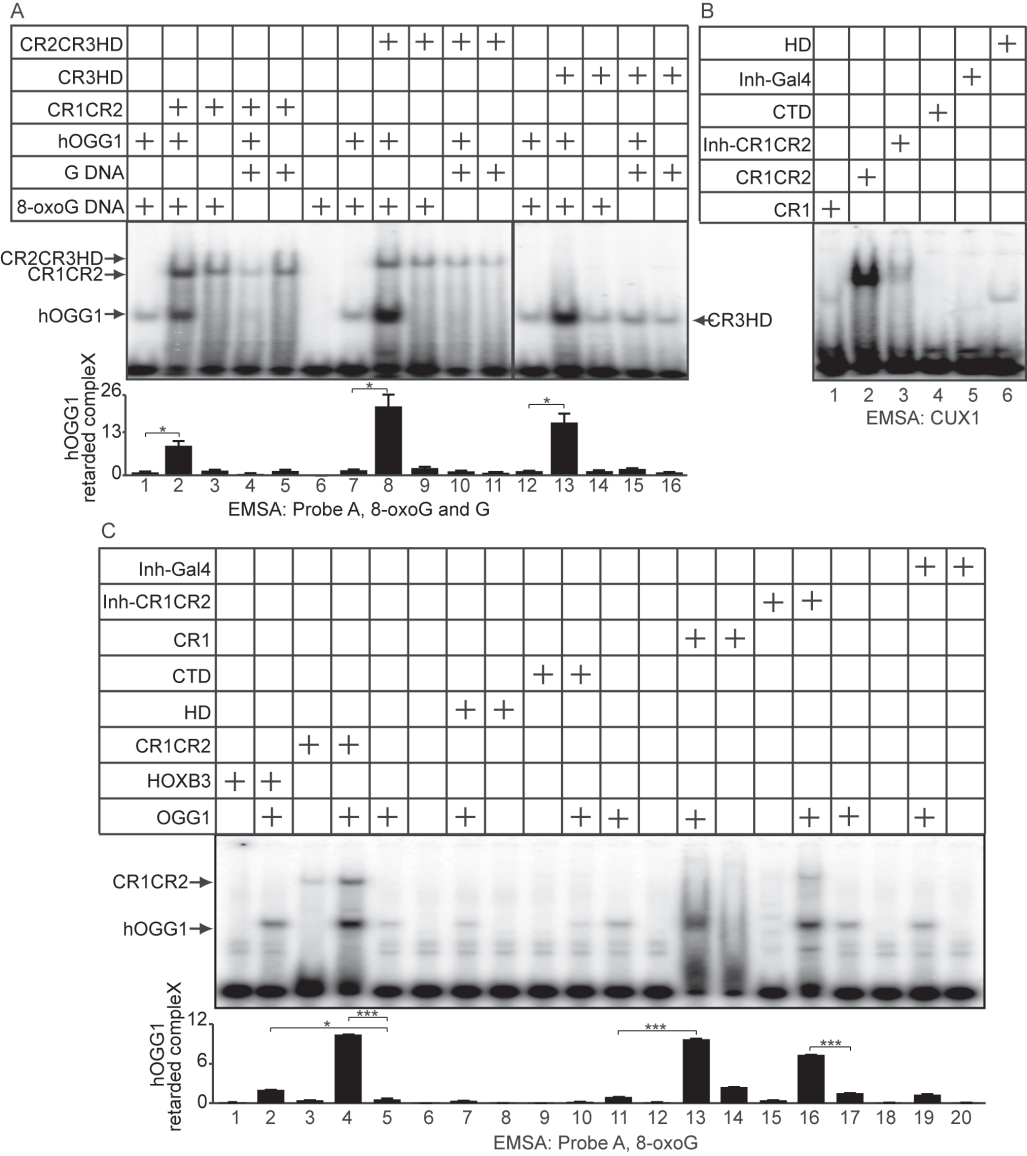
DNA Binding properties of Cut repeats and OGG1 EMSA was performed using OGG1 and/or bacterially purified proteins, as indicated. **(A)** The oligonucleotides correspond to probe A and contain an 8-oxoG or an unmodified G (Figure S1A). **(B)** The oligonucleotides contain a CUX1 consensus binding sites (Figure S2D). **(C)** The oligonucleotides correspond to probe A and contain an 8-oxoG (Figure S1A).

In the course of the reaction carried out by OGG1 on 8-oxoG-containing DNA, a Schiff-base intermediate is formed [34]. Figure 6 shows that CR1CR2 stimulates Schiff-base formation by OGG1. Since the Schiff-base is formed concomitantly with the excision of the base, these results suggest that Cut repeats may stimulate the glycosylase step or the binding of OGG1 to 8-oxoG-containing DNA. Electrophoretic mobility shift assays (EMSAs) were performed using oligonucleotides that contain an 8-oxoG or a G base (probe A, Figure S3A), and OGG1 in the presence or absence of CR1CR2, CR2CR3HD or CR3HD. Each of these CUX1 recombinant proteins stimulated the binding of OGG1 to the probe containing an 8-oxoG, but not to probe containing a normal G base (Figure 7A, CR1CR2, compare lanes 1 and 2; CR2CR3HD, compare lanes 7 and 8; CR3HD, compare lanes 12 and 13). Note that the CUX1 recombinant proteins also generate a retarded complex, which in the case of CR3HD co-migrates with that of OGG1 (Figure 7A, lane 14). However, the mobility of the complex produced by OGG1 was not altered in the presence of any CUX1 protein, suggesting that OGG1 and CUX1 do not form a ternary complex with DNA. We noted that HOXB3 was also able to stimulate the binding of OGG1 to 8-oxoG DNA, albeit weakly (Figure 7C, lane 2). However, HOXB3 did not stimulate OGG1 enzymatic activity (Figure 5D, lane 9).

### Stimulation of OGG1 *in vitro* does not require high affinity DNA binding by Cut repeats

A single Cut repeat cannot bind to DNA with high affinity when expressed as a monomer [27]. We confirmed these findings using oligonucleotides containing the CUX1 consensus binding site and his-tagged CUX1 proteins containing one or two Cut repeat(s). The CR1 protein generated only a weak retarded complex, whereas the CR1CR2 protein generated a strong retarded complex (Figure 7B, lanes 1 and 2). Yet, CR1 was able to stimulate the binding of OGG1 to probe A (Figure 7C, compare lane 13 with 11), the glycosylase activity of OGG1 (Figure 5B, compare lane 3 with 2), and its AP-lyase activity (Figure 5E, compare lane 5 with 7). To confirm that the ability to bind DNA with high affinity is not required for Cut repeats to activate OGG1, we prepared a protein containing CR1CR2 fused to the N-terminal auto-inhibitory domain CUX1 (Inh-CR1CR2) [35]. The auto-inhibitory domain greatly reduced the binding of CR1CR2 to the CUX1 consensus binding site (Figure 7B, compare lanes 2 and 3). The Inh-CR1CR2 protein did not generate a detectable retarded complex with the 8-oxoG-containing probe A and did not reduce the amount of free probe (Figure 7C, lane 15). Yet, the Inh-CR1CR2 protein was still able to increase the binding of OGG1 to 8-oxoG DNA and stimulate its enzymatic activity (Figure 7C, compare lane 16 with 11; Figure 5B, compare lane 5 with 2). In summary, these results demonstrate that a single Cut repeat is sufficient to stimulate several biochemical activities of OGG1 and that the stimulation of OGG1 can be dissociated from the high affinity DNA binding property of Cut repeats.

### The proliferation block of Cux1^−/−^ MEFs in 20% oxygen is rescued by CUX1 and by the Cut repeats 1 and 2

**Figure 8:**
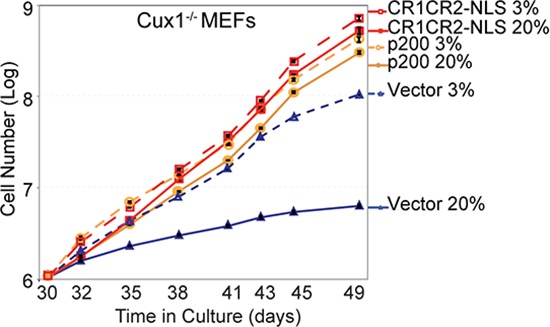
The proliferation block of Cux1−/− MEFs in 20% oxygen is rescued by CUX1 and by the Cut repeats 1 and 2 Cux1^−/−^ MEFs were stably infected in 3% oxygen with a retrovirus expressing p200 CUX1, CR1CR2-NLS or an empty vector. Following selection, cells were maintained in 3% or 20% oxygen and counted over a period of 19 days, from days 30 to 49.

Having established that ectopic expression of recombinant CUX1 proteins can restore efficient repair of oxidative DNA damage in Cux1^−/−^ MEFs (Figure 2), we next investigated whether the proliferation block in 20% oxygen could also be rescued. When switched to 20% oxygen, Cux1^−/−^ MEFs carrying the empty vector divided no more than two to three times between days 30 and 49 (Figure 8). While this rate of proliferation is very modest, it is already better than what we observed we earlier cultures of Cux1^−/−^ MEFs (Figure 1A). This is likely due to the adaptive or mutagenic events that eventually enable MEFs to proliferate in 20% oxygen [4]. Strikingly, however, ectopic expression of both p200 CUX1 and CR1CR2-NLS completely rescued the proliferation defect of Cux1^−/−^ MEFs in 20% oxygen (Figure 8). Together, results presented in Figures 2 and 8 show that ectopic expression of recombinant CUX1 proteins in Cux1^−/−^ MEFs prevents the gradual increase in DNA damage and restores the capacity to proliferate in 20% oxygen.

## DISCUSSION

RNAi-mediated knockdown and gene inactivation demonstrated that CUX1 is required for efficient repair of oxidative DNA damage, while base excision repair assays established that Cut repeat domains can increase the binding of OGG1 to 8-oxoG-containing DNA and stimulate both its glycosylase and AP-lyase activities (Figures 1, 3, 5 and 6). Based on these results, we propose that the p200 CUX1 protein serves as an accessory factor in the sub-pathway of base excision repair that removes oxidized purines.

Cellular senescence is believed to function as a tumor suppression mechanism, yet the senescence-associated secretory phenotype can promote cancer development and progression by stimulating the proliferation of tumor cells, inducing an epithelial-to-mesenchymal transition or protecting neighboring cells from the effects of chemotherapy [1]. Similarly, CUX1 belongs to this class of cancer genes that can both protect against cancer and promote cancer development and progression depending on physiological context [14]. It remains to be demonstrated whether the DNA repair function of CUX1 contribute to its role as a tumor suppressor, however, it is clear that RAS-driven cancer cells exploit this function of CUX1 to avoid senescence and continue to proliferate in spite of elevated levels of reactive oxygen species [16].

The finding that Cux^−/−^ MEFs accumulate DNA damage and exhibit a drastic proliferation defect in 20% oxygen suggests that the role of CUX1 in base excision repair will be particularly important in situations of oxidative stress and in cell types that consume more oxygen. Neurons have very high rates of oxygen metabolism due to their high glucose requirement and the dependence on aerobic oxidation of glucose as their source of energy [36]. Indeed, experimental evidence indicates that defective base excision repair can promote post-mitotic neuronal cell death and neurodegenerative disease [9, 37, 38]. In particular, defective base excision repair was demonstrated in brain from individuals with Alzheimer's disease and amnestic mild cognitive impairment [39]. Interestingly, gene duplication during evolution led to the existence of two CUX genes. While CUX1 is expressed ubiquitously, CUX2 exhibits neural-specific expression [40]. Future studies should investigate the status of CUX1 and its neuron-specific homolog, CUX2, in the aging brain and evaluate the pertinence of therapeutic intervention employing drugs that mimic the effect of Cut repeats on OGG1.

We can envisage a number of mechanisms by which Cut repeats stimulate the binding of OGG1 to DNA that contains an 8-oxoG lesion, in the absence of a high affinity CUX1 binding site (Figure 7). Cut repeats may bind to 8-oxoG residues long enough to promote cooperative binding with OGG1, however, we did not observe any retarded complex that corresponds to a ternary complex involving DNA, OGG1 and CUX1 (Figure 7A and 7C). It is possible that Cut repeats transiently alter the conformation of DNA to facilitate the recognition of 8-oxoG by OGG1 or, as suggested from previous studies, help OGG1 distort DNA while searching for damaged bases [41]. A third possibility, that does not exclude the others, is that Cut repeats induce an allosteric change in OGG1 itself. This notion is consistent with structural studies reporting conformational transitions of OGG1 associated with its biochemical activities ([42–45], reviewed in [6]). Assuming it would be possible to trap a Cut repeat-OGG1 complex, structural analysis might be able to provide important insights on the mechanism by which 8-oxoG lesions are recognized and excised in cells. Moreover, we suggest that it should be possible to identify small molecules that reproduce the effects of Cut repeats on OGG1. Indeed, previous studies using oligonucleotide substrates containing a specific lesion and attached to a fluorescent moiety have established the feasibility of performing high throughput screening assays [46, 47].

The discovery that CUX1 plays a role in base excision repair by stimulating the function of OGG1 has important implications beyond the repair of 8-oxoG lesions. The possibility that the function of DNA glycosylases could be facilitated by DNA binding proteins has not previously been thoroughly investigated. Indeed, in short-lived organisms with a small genome there is probably no need for ancillary proteins in base excision repair. In organisms with a large genome, however, we can envisage the benefit of having transcription factors participate in the maintenance of genome integrity. As their primary role is to scan the genome to determine which regions should be expressed and which ones should remain silent, transcription factors are well positioned to help in the recognition and repair of altered bases. In support of this notion, the YB-1 transcription factor has previously been reported to stimulate the base excision activity of NEIL2 and NTH1 [48, 49], while the architectural transcription factor HMGB1 was shown to stimulate the strand incision activity of APE1 [50]. The precedents set by CUX1, YB-1 and HMGB1 will justify further investigations into distinct classes of DNA binding proteins that participate in the repair of specific types of base damage. We note that the mammalian genome contains several hundred coding sequences for DNA binding domains, several of which with ill-defined transcriptional functions. We speculate that some DNA binding proteins that are present at more than 100,000 molecules per cell, like CUX1, function in processes other than transcription.

## MATERIALS AND METHODS

### Cell culture and virus production

Primary mouse embryonic fibroblasts (MEFs) were grown at 37°C, 5% CO_2_ and 3% O_2_ unless otherwise indicated. Lentiviruses and retroviruses were produced as previously described [16].

### Bacterial protein expression

Expression of his- or GST-tagged fusion proteins containing CUX1 peptides [27], peroxisome proliferator-activated receptor δ (PPARδ, Addgene plasmid 16548 [51]), transcription factor 4 DNA binding domain (TCF DBD, Addgene plasmid 16488 [52]), homeodomain protein B3 (HOXB3, Addgene plasmid 8524 [53]) was induced with isopropyl-β-D-thiogalactopyranoside in the BL21 strain of *Escherichia coli*. In the case of his-tagged fusion proteins to be used with OGG1, several buffer exchanges were carried in 3-kDa molecular weight cut-off dialysis membrane (Amicon Ultra, Millipore) to bring down imidazole concentration to less than 0.1 μM.

### Cell proliferation

CUX1^+/+^, Cux1^−/−^ MEFs and Cux1^−/−^ MEFs stably expressing p200, CR1CR2 or carrying an empty vector were plated in either 3% O_2_ or atmospheric O_2_ at a density of 5 × 10^5^ cells in a T75 flask. Cells were trypsinized and counted on a hemocytometer at every passage. Each time point was done in triplicate, and the averages ± standard deviations were calculated. Experiments were repeated three times, and a representative experiment is shown.

### Cell viability assays

5 × 10^4^ CUX1^+/+^ and Cux1^−/−^ MEFs were cultured in 3% O_2_ and incubated in medium containing various concentration of H_2_O_2_ (Sigma) for 60 min, and then cultured in fresh medium for another 24 h. Cells were trypsinized and counted on a hemocytometer in the presence of trypan blue. Only unstained living cells were counted. Experiments were repeated three times, each concentration of oxidizing agents were done in triplicate, and the averages ± standard deviations were calculated.

### Single-cell gel electrophoresis

For H_2_O_2_ treatment, cells at ~80% confluence were treated with 50 μM H_2_O_2_ on ice for 20 min. After treatment, cells were allowed to recover at 37°C in fresh medium for the indicated periods of time before harvesting. Comet assays were carried out using pre-coated slides according to manufacturer protocol (Trevigen, MD, USA). The slides were stained with propidium iodide and visualize with Axiovert 200M microscope with an LSM 510 laser module (Zeiss). Comet tail moments were measured on a minimum of 50 cells using the CometScore software (TriTeck Corp).

### *In vitro* binding assay

Bacterially expressed his-tagged CUX1-CR1CR2 and HOXB3 proteins as well as proteins from bacteria carrying the empty his-tag vector were bound to Dynabeads magnetic beads (Life technologies) and incubated overnight with 100 ng of purified GST-OGG1 proteins. As indicated, some binding assays were performed on lysates pretreated with 65 U/ml of benzonase (Novagen) for 15 min at 37°C or in the presence of 100 μg/ml of ethidium bromide. The samples were washed five times and separated by SDS-PAGE followed by immunoblotting with anti-OGG1 antibody.

### *In vitro* 8-oxoG cleavage assay

Cleavage reactions with bacterially purified proteins were conducted using 50 nM hOGG1 (New England Biolabs, Ipswich, MA), and 50 nM of BSA or the indicated proteins, unless otherwise indicated, in 25 mM NaCl, 10 mM Tris (pH 7.5), 1 mM MgCl_2_, 5 mM EDTA (pH 8.0), 5% glycerol, 1 mM of DTT and 1 pmol of ^32^P-radiolabeled double-stranded oligonucleotides containing an 8-oxoG base (Figure S3A). Note that when using his-tagged fusion proteins, it is important at the end of the purification to carry several buffer exchanges in order to reduce imidazole concentration. In our hands, OGG1 is stimulated by imidazole at concentrations above 0.4 mM. Reactions with total cell extracts were performed as described by Paz-Elizur et al. [54] with slight modification. Briefly, 20 μg of total proteins and 0.5 pmol of ^32^P radiolabeled oligonucleotides were used with 100 ng of poly(dI-dC) as a nonspecific competitor DNA. In both assays, cleavage reactions were performed at 37°C as previously described. The DNA was loaded on a pre-warmed 20% polyacrylamide-urea gel (19:1) and separated by electrophoresis in Tris-borate and EDTA (TBE; pH 8.0) at constant 20 mAmp.

### Electrophoretic mobility shift assay (emsa)

EMSAs were performed as previously described [25], using Probe A (Figure S3A) or CUX1 consensus sequence (5′-TCGAGAAATGAAGCTTATCGATATCGTCTCGA-3′). 50 nM of bacterially purified proteins were used in the reaction together with 60 ng of poly(dI-dC) as a nonspecific competitor DNA.

### Sodium borohydride trapping of hogg1

5′-end-labeled 32-mer duplex containing a 8-oxoG (50 nM) was incubated with hOGG1, and CR1CR2 or BSA at the indicated concentrations. After incubation at 37°C for 30 mins, 50 mM sodium borohydride was added and the reactions were pursued for another 15 min at 37°C. The reaction was stopped in SDS sample loading buffer and heated for 5 min at 100°C. The trapped complexes were separated from free substrate by 10% SDS-PAGE gel.

## SUPPLEMENTARY FIGURES


