# Supplementary material for: The function of cux1 in oxidative dna damage repair is needed to prevent premature senescence of mouse embryo fibroblasts
Source: Oncotarget. 2015 Feb 17;6(6):3613–26. doi: 10.18632/oncotarget.2919 (PMC4414141; doi:10.18632/oncotarget.2919)
Supplement: Supplementary file 1 [file oncotarget-06-3613-s001.pdf]

SUPPLEMENTARY FIGURES

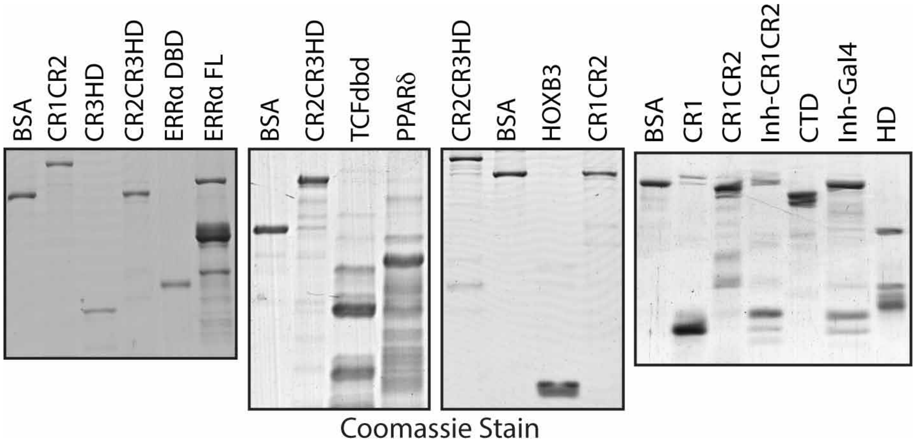

Supplementary Figure S1: Coomassie stains of bacterially expressed recombinant proteins. Recombinant proteins were expressed in bacteria, purified by affinity chromatography, separated by SDS-PAGE and stained with coomassie blue.

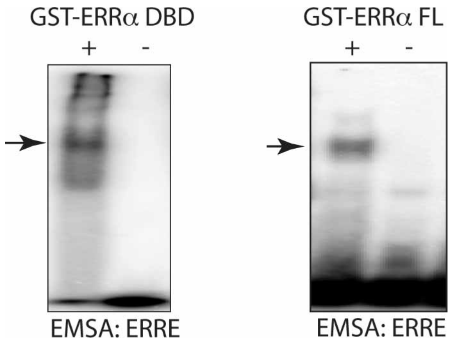

Supplementary Figure S2: GST-ERRα DNA binding domain and GST-ERRα full-length are functional as judged from DNA binding assays. EMSA was performed using bacterially purified GST-ERREα DNA binding domain (DBD) and full length protein (ERREα FL) and oligonucleotides containing the ERREα consensus binding site, 5′-GCGTCCAGATGAAGATCAAAGAA-3′.

A

Probe A :

```
5'-*CCGGTGCATGACACTGTXACCTATCCTCAGCG-3'
3'- GGCCACGTACTGTGACACTGGATAGGAGTCGC-5'
where X = 8-oxoG or G
32 nt substrate:
   *CCGGTGCATGACACTGTXACCTATCCTCAGCG
17 nt product:
   *CCGGTGCATGACACTGTX
```

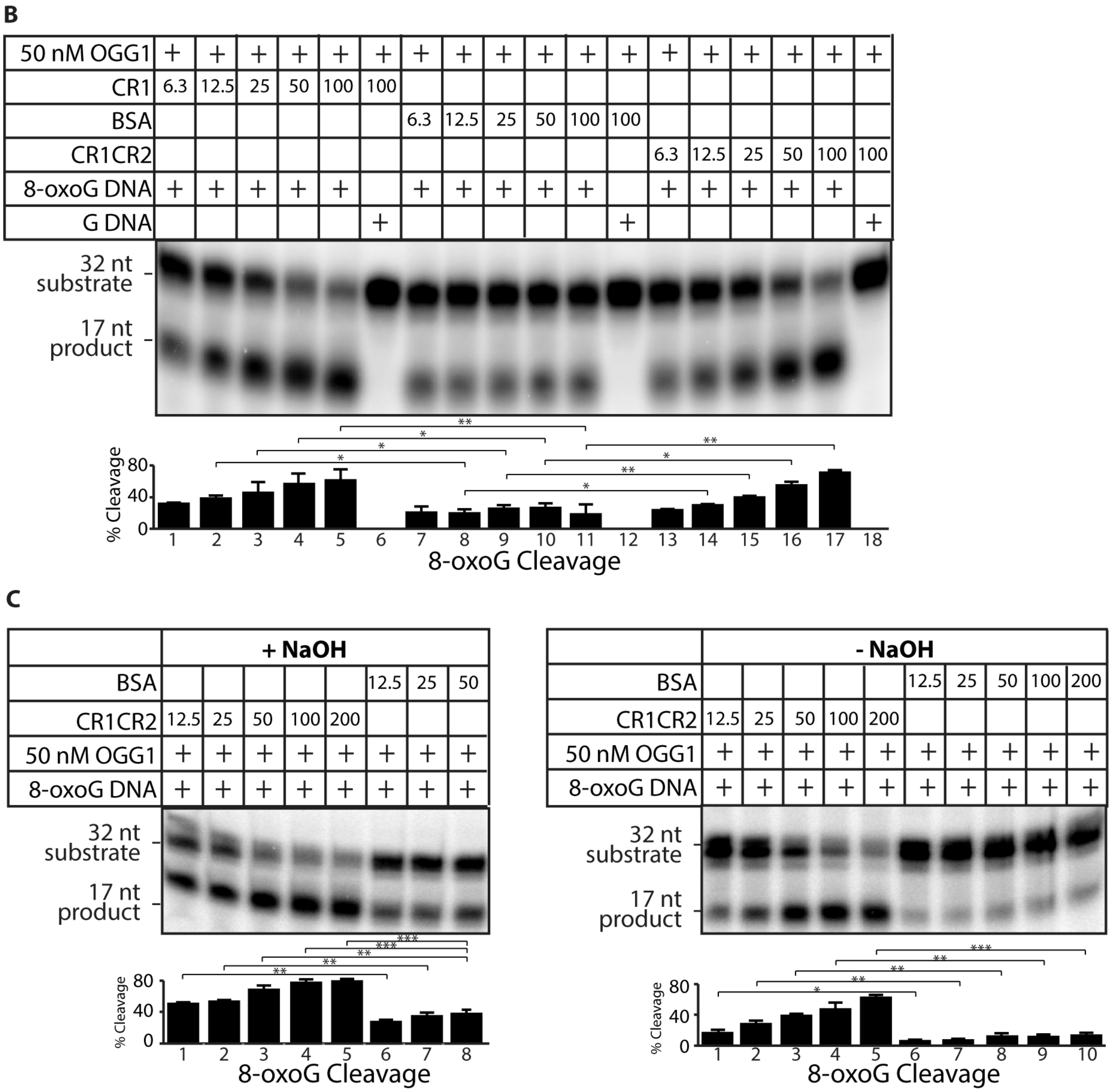

Supplementary Figure S3: Increasing concentration of Cut repeats lead to higher OGG1 glycosylase and AP-lyase activities. (A) Probe A: double-stranded oligonucleotides containing an 8-oxoG or an unmodified G at the X position were labeled with 32P-gamma ATP at the 5’ end of the top strand (*) using polynucleotide kinase. The probe sequence is identical to that used in (8). Note that the sequence does not contain a consensus binding site for CUX1. **(B)** The 8-oxoG cleavage assay was performed using 50 nM of OGG1 and increasing amounts (nM) of BSA, CUX1 CR1 or CR1CR2. No OGG1 was added in the reactions of lanes 6, 12 and 18 to verify that BSA or CUX1 proteins alone do not cleave the 8-oxoG probe. **(C)** The 8-oxoG cleavage assay was performed using 50 nM of OGG1 and increasing amounts (nM) of CUX1 CR1CR2 . After 30 min incubation at 37°C, reactions were stopped, and DNA was submitted to treatment with NaOH (+NaOH) or not (–NaOH). Reactions in the presence of NaOH monitor OGG1 glycosylase activity only, whereas reactions in the absence of NaOH reveal OGG1 glycosylase and AP-lyase activities.

A

Probe B:

```
5'-*GCGTCCAGATGAAXATCAAAGAACTT-3'
3'- CGCAGGTCTACTTCTAGTTTCTTGAA-5'
where X = 8-oxoG
26 nt substrate:
   *GCGTCCAGATGAAXATCAAAGAACTT
13 nt product:
   *GCGTCCAGATGAAX
```

B

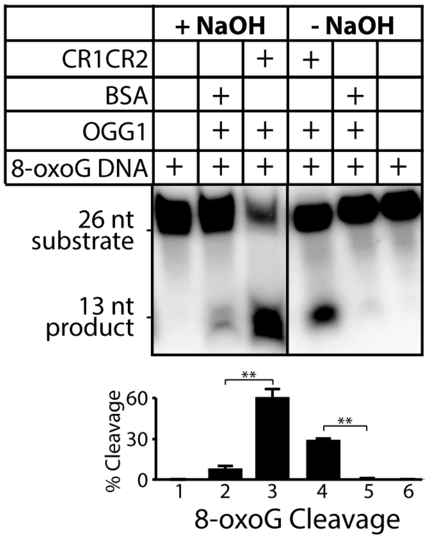

Supplementary Figure S4: CR1CR2 stimulates the glycosylase and AP-lyase activity of OGG1 on a different DNA sequence. **(A)** Double-stranded oligonucleotides containing a different sequence with an 8-oxoG or an unmodified G at the X position were labeled with 32P-gamma ATP at the 5' end of the top strand (*) using polynucleotide kinase. Note that the sequence does not contain a consensus binding site for CUX1. **(B)** The 8-oxoG cleavage assay was performed using 50 nM of OGG1 and 50 nM of BSA and CUX1 CR1CR2 using Probe B in the absence and presence of NaOH.

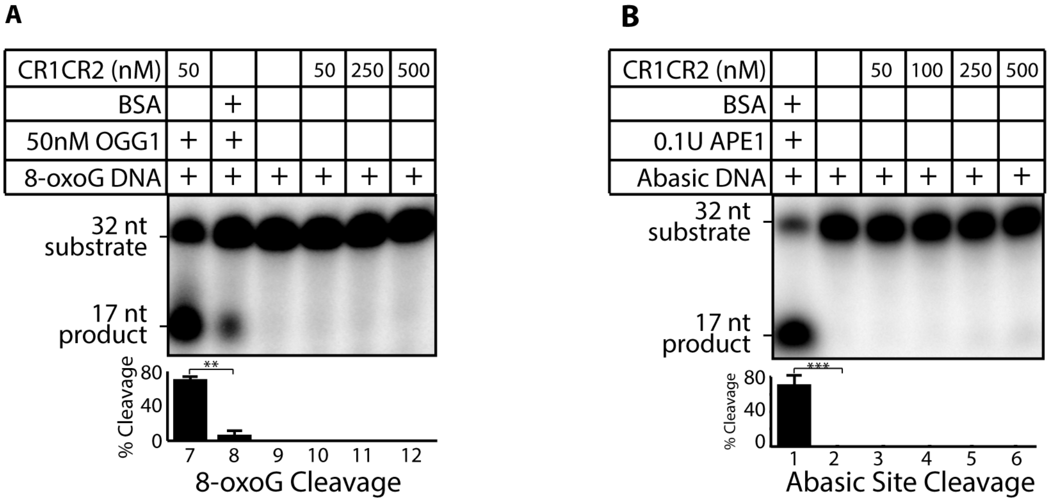

Supplementary Figure S5: CR1CR2 alone does not cleave at 8-oxoG or abasic sites. **(A)** The 8-oxoG cleavage assay was performed using increasing amount of CUX1 proteins, CR1CR2, to verify that CUX1 proteins alone do not cleave the 8-oxoG probe (Figure S3A). As controls, the reaction was carried with purified OGG1 together with BSA or CR1CR2, as indicated. **(B)** Cut repeats cannot cleave at an abasic site. The probe is identical to probe A (Figure S3A), except that the 8-oxoG base was replaced for an abasic site. The cleavage assay was performed for 30 min at 37°C with the indicated proteins. The results demonstrate that CR1CR2 is unable to cleave at the abasic site.
